# Druggable target ATAD2 enhances the malignant progression and cooperates with E2F1 to up-regulate PDK1 expression in glioma

**DOI:** 10.1016/j.gendis.2025.101810

**Published:** 2025-08-14

**Authors:** Shenghua Zhuo, Liangwang Yang, Zhimin Chen, Shenbo Chen, Shuo Yang, Taixue Chen, Wen-Shu Wu, Kai Wang, Kun Yang

**Affiliations:** a International Center for Aging and Cancer, Department of Neurosurgery, The First Affiliated Hospital of Hainan Medical University (Hainan Academy of Medical Sciences), Haikou, Hainan 571199, China; b Department of Medical Oncology, Shanghai Pulmonary Hospital, Tongji University Medical School Cancer Institute, Tongji University School of Medicine, Shanghai 200433, China

**Keywords:** ATAD2, Cancer-testis antigen, E2F1, Glioma, PDK1

## Abstract

Gliomas are characterized by high mortality and disability rates. Cancer-testis antigens (CTAs) are among the most promising therapeutic targets for combating cancer. While several CTAs have been associated with the development and progression of gliomas, the role of ATPase family AAA domain-containing protein 2 (ATAD2) in this context has not been thoroughly investigated. In this study, both *in vitro* and *in vivo* experiments validated the role of ATAD2 in enhancing malignant phenotypes. The LN229 cell lines were employed for RNA-seq and proteomics to uncover downstream targets of ATAD2. Results showed that elevated ATAD2 expression was noted in glioblastoma (GBM). ATAD2 knockdown significantly reduced the proliferation, migration, and invasion capabilities of GBM cells, while its overexpression had the opposite effect. The knockdown of ATAD2 led to a decrease in subcutaneous tumor size and weight, a reduction in Ki67 expression, and an extension of survival in mice bearing intracranial *in situ* tumors. Mechanistically, a positive feedback loop involving ATAD2 and E2F transcription factor 1 (E2F1) was identified to enhance the transcriptional activation of pyruvate dehydrogenase kinase 1 (PDK1). Notably, the expression levels of these genes were found to be positively correlated, with patients exhibiting high levels of these genes tending to have poorer prognoses. These findings demonstrate that ATAD2 plays a pivotal role in the malignant progression of glioma and synergizes with E2F1 to promote PDK1 expression, suggesting its potential as a therapeutic target for glioma.

## Introduction

Gliomas constitute 26.3% of all central nervous system tumors, with glioblastoma (GBM) accounting for 50.9% of malignant brain tumors.^1^ The limited treatment options for gliomas, along with their high mortality and disability rates, impose a significant burden on healthcare systems and society.^2^ Researchers are actively pursuing highly sensitive, specific, and transformative therapeutic targets to combat this malignancy.

Tumor-associated antigens present promising therapeutic targets, including cancer germline antigens, human endogenous retroviruses, tissue differentiation antigens, and overexpressed antigens.^3^ Cancer germline antigens, also known as cancer-testis antigens (CTAs), form a distinct subset with specific expression profiles.^4^ CTAs are involved in various oncogenic signaling pathways that promote tumor growth and inhibit apoptosis.^5^^,^^6^ Typically restricted to germ cells, CTAs can be reactivated in cancers, and their immunogenicity underscores their potential in tumor therapy.^7^, ^8^, ^9^ Numerous clinical trials targeting CTAs are currently in progress.^10^ Our recent review summarized that CTAs are expressed at variable levels in gliomas, with elevated levels of certain CTAs being associated with patient prognosis and are highly promising therapeutic targets.^11^ The functional roles and molecular regulatory mechanisms of key CTA genes in glioma remain inadequately characterized.

Among CTAs, ATPase family AAA domain-containing protein 2 (ATAD2, also known as CT137) functions as a transcription factor co-activator and contributes to oncogenesis through multiple pathways.^12^ *ATAD2* is recognized as a pivotal gene in the regulation of melanoma; its knockout in zebrafish melanoma models impedes cellular transformation and tumor tumorigenesis, while its reintroduction restores cancerous transformation capabilities.‍^13^ These findings highlight ATAD2's potential as a therapeutic target in various cancers. A previous study has shown that polo-like kinase 4 influences the proliferation of GBM cells, with exogenous ATAD2 overexpression significantly increasing its expression.^14^ While current research on ATAD2 has primarily focused on other tumor types, its expression patterns, functional roles, and regulatory mechanisms in glioma remain poorly understood.

To explore the role of CTA-related genes in glioma, bioinformatics analysis was employed for clustering and the construction of risk scores. The ATAD2 enzyme, recognized for its bioactive compounds and druggable structure, was identified as a promising therapeutic target. Its expression level in glioma was evaluated, and its influence on malignant phenotypes, particularly proliferation, was examined through cell cultures and animal studies. The regulatory mechanisms of ATAD2 were further investigated using integrated RNA-seq, proteomics analysis, and dual luciferase reporter assays.

## Material and methods

### Clinical specimens and animals

Paraffin-embedded pathological tissue samples and relevant clinicopathological data were collected from six patients whose non-pathological brain tissues were removed during surgery for brain trauma, as well as 28 patients diagnosed with astrocytoma, 35 with oligodendroglioma, and 63 with GBM, as diagnosed by the pathology department. Female BALB/c nude mice, aged 4–6 weeks and weighing 18–20 g, were sourced from the Guangdong Medical Experimental Animal Center (Guangzhou, China) and maintained under specific pathogen-free conditions. The mice were housed in standard cages at 23 °C, under a 12-h light–dark cycle, and were provided with unlimited access to food and water.

### Data sources

RNA-seq data (fragments per kilobase of exon model per million mapped fragments, FPKM values) were sourced from the Chinese Glioma Genome Atlas (CGGA) (mRNAseq_693, mRNAseq_325, http://www.cgga.org.cn/)^15^ and The Cancer Genome Atlas (TCGA-LGG (low-grade glioma) and TCGA-GBM, https://portal.gdc.cancer.gov/). Batch correction was performed using the ComBat algorithm, which is part of the “sva” software package.^16^ Clinical information for TCGA was extracted from previously published studies.^17^ The Rembrandt cohort data^18^ were retrieved from the “Other data” section of the CGGA database. The CGGA dataset was used as the training cohort, while the TCGA and Rembrandt datasets served as validation cohorts. A total of 255 CTA genes were identified from the CTdatabase (http://www.cta.lncc.br/),^19^ and 237 from GeneCards (https://www.genecards.org/).^20^ Among these, 212 genes were found in both databases, yielding a total of 280 CTA genes. Subsequently, 93 CTA genes with available expression data in the CGGA dataset were chosen for further analysis (Table S1).

### Bioinformatics analysis

Non-negative matrix factorization (NMF)^21^ was utilized to classify CGGA glioma patients. Before this analysis, Cox regression analysis was performed using the R package “survival” to identify candidate CTA-related genes associated with overall survival (OS). A total of 69 CTA-related genes that correlated with prognosis were selected for patient clustering.

To assess the prognostic significance of these CTA genes, univariate Cox regression analysis was conducted across all three datasets, applying a significance threshold of *P* < 0.05 and hazard ratio (HR) greater than 1 to indicate a correlation between elevated gene expression and poor prognosis. Genes that were consistently linked to poor prognosis from the three cohorts were selected for further analysis. Following established methodologies,^22^ the risk score, termed CTARS, was calculated using LASSO-Cox regression with the R package “glmnet”,^23^ resulting in the final selection of 10 genes and their corresponding coefficients. CTARS = (0.365293477 × *KIF2C* expression level) + (−0.190865526 × *ATAD2* expression level) + (0.043627184 × *IL13RA1* expression level) + (0.24297281 × *IGF2BP3* expression level) + (0.030737919 × *SPAG1* expression level) + (0.018849277 × *SPA17* expression level) + (0.053422415 × *SPAG4* expression level) + (0.208430977 × *ANKRD45* expression level) + (0.10665032 × *IL13RA2* expression level) + (−0.034270644 × *MAEL* expression level). Patients were categorized into low or high-CTARS groups based on the median score. In both univariate and multivariate Cox regression analyses, the following variables were included as predefined dichotomous categories: gender, isocitrate dehydrogenase (IDH) mutation status, 1p/19q codeletion status, and O-6-methylguanine-DNA methyltransferase promoter (MGMTp) methylation status. Age was stratified as < 40 years versus ≥40 years, tumor grade as WHO IV versus non-WHO IV, and CTARS scores as high versus low groups. Variables showing statistically significant differences (*P* < 0.05) in the univariate analysis were subsequently entered into the multivariate analysis. Validation of CTARS involved applying the derived formula from the training cohort to calculate scores for each patient in the test cohorts. The “RMS” package was used to create a prognostic nomogram that included four independent variables: age, tumor grade, 1p/19q status, and CTARS. Calibration curves for 1-, 3-, and 5-year prognostic estimates were used to assess the congruence between the predicted and observed survival rates, followed by validation using two independent cohorts.

Druggability analysis was performed using the DepMap database (https://depmap.org/portal/), while the GEPIA (http://gepia.cancer-pku.cn/)^24^ and UALCAN (https://ualcan.path.uab.edu/index.html)^25^ databases were utilized to analyze the expression levels of *ATAD2* mRNA and protein in normal versus glioma tissues. The CPTAC_GBM dataset from the LinkedOmics database (http://www.linkedomics.org/admin.php)^26^ was employed to investigate transcription factors associated with ATAD2 expression. Correlation analysis of the expression of multiple mRNAs in public glioma cohorts was conducted using the GlioVis database (http://gliovis.bioinfo.cnio.es/).^27^ The GSE138794, GSE103224, and GSE131928_10X datasets from the TISCH2 database (http://tisch.comp-genomics.org/) were utilized to analyze the single-cell level expression of target genes in glioma.^28^

### Cell lines, culture, and reagents

The LN229, U251MG, and T98G cell lines were obtained from Pricella (Wuhan, China), the HA1800 and U118MG cell lines were acquired from Jenniobio (Guangzhou, China), and the U87 cell line was sourced from Servicebio (Wuhan, China). All the cell lines were authenticated through short tandem repeat analysis and screened for mycoplasma contamination to ensure that they were negative. The cells were maintained at 37 °C in an incubator with 5% CO_2_ and cultured in DMEM high-glucose medium (Gibco, cat# C11995500BT) supplemented with 1% (v/v) penicillin/streptomycin (Gibco, cat# 15140-122) and 10% (v/v) fetal bovine serum (Sigma, cat# F8318).

### Lentivirus preparation and stable cell line generation

For the construction of *ATAD2* shRNA expression vectors, the following sequences were utilized: Sh-1: GCCTAATTGATGTAGTATGAA, Sh-2: CCAGAGTGCAAGTCATGATTT, and Sh-3: GTAGGATTAGAAGTCGTTATA. These shRNA sequences were incorporated into the pHBLV vector, while *ATAD2* cDNA was cloned into the CMV-ZsGreen1 vector. ATAD2 overexpression plasmids and shRNA plasmids, as well as lentiviral packaging vectors, were sourced from Bioegene (Shanghai, China). The LN229 and U251MG cell lines were utilized for ATAD2 knockdown, and the U118MG cells were employed for ATAD2 overexpression. Infection was carried out using LV-Enhance, an infection-enhancing reagent, at a multiplicity of infection of 10. Stable transfection was achieved by selecting cells with puromycin (2.5 μg/mL) for two weeks.

### Plasmid and siRNA transfection

E2F transcription factor 1 (E2F1) siRNA, along with negative, positive, and fluorescence control siRNAs, as well as the E2F1 overexpression plasmid (pcDNA3.1-E2F1-3FLAG) and its control plasmid, were obtained from Bioegene (Shanghai, China). The specific *E2F1* siRNA sequences are as follows: Si-1: CTACTCAGCCTGGAGCAAGAA, Si-2: CGCTATGAGACCTCACTGAAT, and Si-3: CGTGGACTCTTCGGAGAACTT. Transfection was conducted according to the manufacturer's instructions using the jetPRIME® *in vitro* DNA & siRNA transfection reagent (Polyplus, France).

### Cell viability assay

A total of 2000 cells were seeded into each well of a 96-well plate, with three replicate wells for each experimental condition. At each time point (4-h adhesion period, 0, 1, 2, and 3 days), the culture medium in the test wells was removed, and 200 μL of fresh medium containing 10 μL of CCK-8 solution (NCM, cat# C0005) was added. The plate was then incubated for 1 h, after which the optical density (OD) at 450 nm was measured using a multimode microplate reader (TECAN, cat# Spark).

### Colony formation assay

For the colony formation assays, 1 × 10^3^ cells were seeded per well in a 6-well plate. After a 10 to 14-day culture period to allow for the formation of single colonies, the cells were then fixed with methanol and stained with 0.5% crystal violet (Biosharp, cat# BS94A). Once dried, the culture plate was scanned and photographed, and the number of colonies was counted.

### Transwell migration and invasion assays

The assays were conducted using a 24-well plate and Transwell chambers with 8.0 μm pore size (LABSELECT, cat# 14342). For the invasion assay, the chamber was pre-coated with Matrigel (Corning, cat# 356234). After a 16-h serum starvation period, 200 μL of a cell suspension in serum-free medium (1 × 10^5^ cells/mL) was added to the upper chamber, while 0.5 mL of medium containing 10% fetal bovine serum (FBS) was placed in the lower chamber. Following a 24-h incubation, migrating or invasive cells that adhered to the bottom of the filter membrane were fixed with methanol, stained with 0.5% crystal violet, and analyzed using an inverted microscope (Nikon, Japan).

### Xenograft tumor growth assays

For the subcutaneous transplantation tumor experiment, LN229 Shco and Sh-3 cells were suspended in phosphate-buffered saline (PBS) at a concentration of 5 × 10^7^ cells per 1.0 mL and mixed with Matrigel at a 1:1 ratio on ice. Six nude mice were randomly assigned, and 0.2 mL of the cell mixture was injected subcutaneously into the lower back, with the left side serving as the Shco group and the right side as the Sh-3 group. The mice were euthanized using carbon dioxide when the transplanted tumors reached 6 weeks and its diameter did not exceed 2.0 cm. The tumors were then excised, photographed, weighed, and their dimensions were measured. The tumor volume was calculated using the formula (length × width^2^)/2, and the tumors were fixed in 4% paraformaldehyde (PFA) for further experimentation.

For the orthotopic intracranial xenograft experiments, the mice were anesthetized by an intraperitoneal injection of 1.5% pentobarbital sodium (0.05 mL/10 g body weight). LN229 Shco or Sh-3 cells (5 × 10^5^) were injected into the right cerebral caudate nucleus using a stereotaxic apparatus and microinjection pump (*n* = 6 per group) following the established protocols.^29^ Daily monitoring included assessments of activity, diet, mental state, and body weight. Euthanasia via carbon dioxide was performed upon the appearance of signs such as weight loss exceeding 20%, decreased appetite, paralysis, persistent inactivity, sustained seizures, stereotyped behavior, or a hunched posture. Specific days of survival and death status were meticulously recorded. Animals that reached 60 days without exhibiting experimental endpoints were documented as surviving for 60 days.

### Western blotting analysis

The cells were lysed on ice for 10 min using radio-immunoprecipitation lysis buffer containing protease inhibitors. The protein concentration was determined using the bicinchoninic acid assay. The samples were then subjected to electrophoresis on sodium dodecyl sulfate‒polyacrylamide gels, followed by transfer to polyvinylidene fluoride membranes. The membranes were blocked with 5% skim milk at room temperature for 1 h and incubated with primary antibodies overnight at 4 °C. The primary antibodies were utilized to detect the following proteins or peptides, respectively: ATAD2 (CST, cat# 50563, 1:2000), β-actin (Servicebio, cat# ZB15001-HRP-100, 1:10000), Flag tag (Proteintech, cat# 66008-4-Ig, 1:5000), pyruvate dehydrogenase kinase 1 (PDK1, ZENBIO, cat# R381931,1:1000), hexokinase 2 (HK2, PTMBIO, cat# PTM-5371, 1:1000), glucose transporter 1 (GLUT1, PTMBIO, cat# PTM-5203, 1:1000), monocarboxylate transporter 4 (MCT4, ZENBIO, cat#862212, 1:1000), and E2F1 (PTMBIO, cat# PTM-6630, 1:1000). The membranes were washed three times with Tris-buffered saline containing 0.1% Tween 20 (TBST) for 5 min each before incubation with HRP-conjugated secondary antibodies from Biosharp (cat# BL001A or BL003A, 1:5000) at room temperature for 1 h, followed by three washes with TBST for 10 min each.

The results were visualized using chemiluminescence on a ChemiDoc MP imaging system (Bio-Rad, USA). For reprobing, the membranes were incubated with β-actin at room temperature for 1 h or with primary antibodies from different species overnight at 4 °C after completely inactivating HRP with 10% acetic acid, as previously reported.^30^ Grayscale analysis was conducted using ImageJ software.

### Hematoxylin-eosin (HE) and immunohistochemistry (IHC) staining

Sections of approximately 5 μm thick were prepared from tissues embedded in paraffin wax. HE staining was conducted using hematoxylin from Sigma (cat# H3136), followed by differentiation in 0.7% hydrochloric acid ethanol from Runnerbio (cat# Bry-0001-03), blue reversion in alkaline solution from Runnerbio (cat# Bry-0001-04), and eosin staining with eosin from Sigma (cat# E4009).

IHC staining, scanning, and scoring were performed according to established protocols.^31^^,^^32^ The samples were classified into low and high expression groups based on an IHC score of less than 6 points. The primary antibodies used for IHC were specific for the following proteins: ATAD2 (CST, cat# 50563, 1:50), Ki67 (Abcam, cat# ab15580, 1:2000), E2F1 (Proteintech, cat# 66515-1-Ig, 1:50), and PDK1 (Proteintech, cat# 18262-1-AP, 1:100).

### Immunofluorescence staining

The cells were fixed on slides (Solarbio, cat# YA0351) using methanol, permeabilized with 0.5% Triton X-100, and blocked with 3% BSA solution. The cells were incubated with an ATAD2 primary antibody (BOSTER, cat# M03855, 1:200), washed with PBS containing 0.1% Tween 20, followed by incubation with a secondary antibody (CoraLite594-Goat Anti-Rabbit, Proteintech, cat# SA00013-4, 1:300). The cells were finally sealed with an anti-fluorescence quenching agent (Biosharp, cat# BL739A) containing DAPI. Imaging was performed using a fluorescence microscope (Nikon, Japan).

### RNA-seq and data analysis

Total RNA was extracted from LN229 Shco and Sh-3 cells utilizing TRIzol reagent (Thermo Fisher, cat# 15596018CN), with three replicates per group, according to the procedure provided by LC-Bio (Hangzhou, China). Standard double-end sequencing was performed on the Illumina NovaSeq™ 6000 platform using PE150 mode, followed by alignment to the human reference genome with HISAT2 and quantification of StringTie genes based on read counts. The assembly, merging, and quantification steps produced original coverage and standardized FPKM values. The analysis utilized read count data and the DESeq2 package, with *P* values derived from a negative binomial distribution model. The Benjamini-Hochberg correction was applied to obtain *q* values. Significance criteria were set at |log2 fold change (FC)| ≥ 1 and *q* value < 0.05.

### Label-free proteomics and data analysis

Protein was extracted from LN229 Shco and Sh-3 cells (three replicates per group). Equal amounts of protein were subsequently analyzed by liquid chromatography-tandem mass spectrometry using a timsTOF Pro mass spectrometry system (Bruker, Germany) at PTM-Bi (Hangzhou, China). The mass spectrometry data were processed using MaxQuant (v1.6.15.0), with quality control performed after data filtering. The iBAQ (*I*) value was determined by dividing the original protein intensity by the theoretical peptide count after library searching. Relative quantification of each protein was calculated using the formula *R*_*ij*_ *=* *I*_*ij*_*/Mean (I*_*j*_*)*. *P* values were determined based on FC and *t*-tests, with a significance threshold set at *P* < 0.05. Proteins were classified as significantly up-regulated if their FC was greater than 1.5, and significantly down-regulated if their FC was less than the reciprocal of 1.5.

### Dual-luciferase reporter assay

pGL3-Basic, pGL3-PDK1 promoter, pcDNA3.1 empty vector, pcDNA3.1-E2F1, pcDNA3.1-ATAD2, and pRL-TK plasmids were co-transfected into LN229 and U251MG cells according to the specific experimental groups. Luminescence from both firefly and Renilla luciferases was separately measured using a multimode microplate reader with a dual luciferase detection kit (Beyotime, cat# RG029S). The reading from Renilla luciferase was used as an internal control to normalize the transfection efficiency.

### Statistical analysis

Statistical analyses and visualizations were performed using R software (v4.0.3) and GraphPad Prism (v9.1.0). The figure legends specify the statistical analysis methods employed and the details of the data presentation. Multivariate Cox regression analysis was used to identify independent prognostic indicators. Receiver operating characteristic (ROC) curve analysis was performed using the “pROC” R package. The clinicopathological characteristics of patients across different subgroups were assessed using the chi-square test. Experiments requiring independent replication were conducted three times, and a *P* value of less than 0.05 was considered statistically significant.

## Results

### Patients with glioma can be classified into two distinct subtypes based on the expression of CTA-related genes

**Figure 1:**
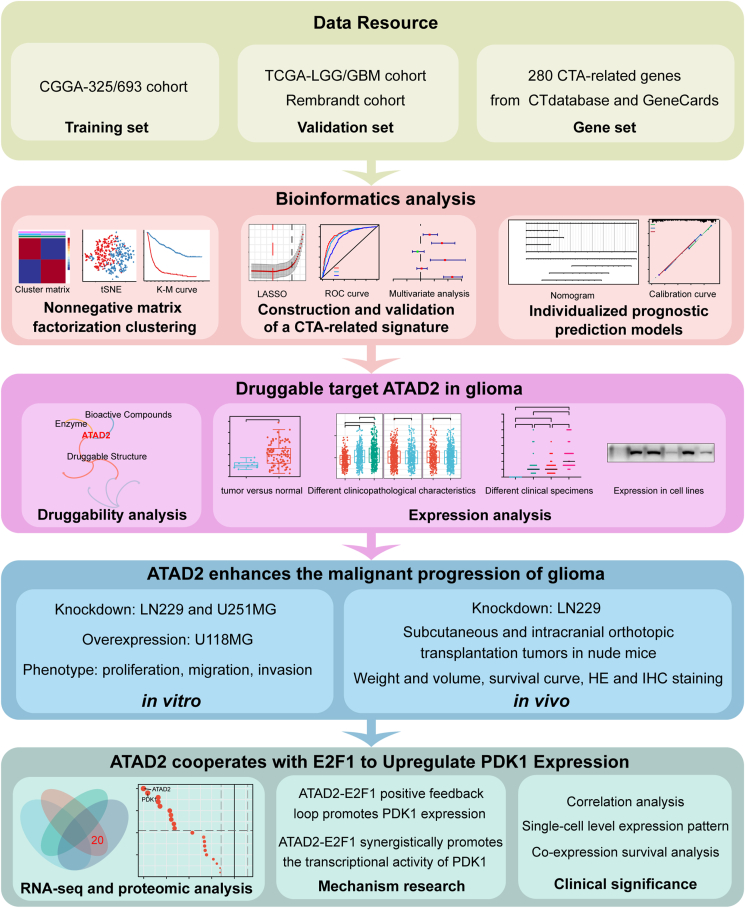
The flowchart of this study.

**Figure 2:**
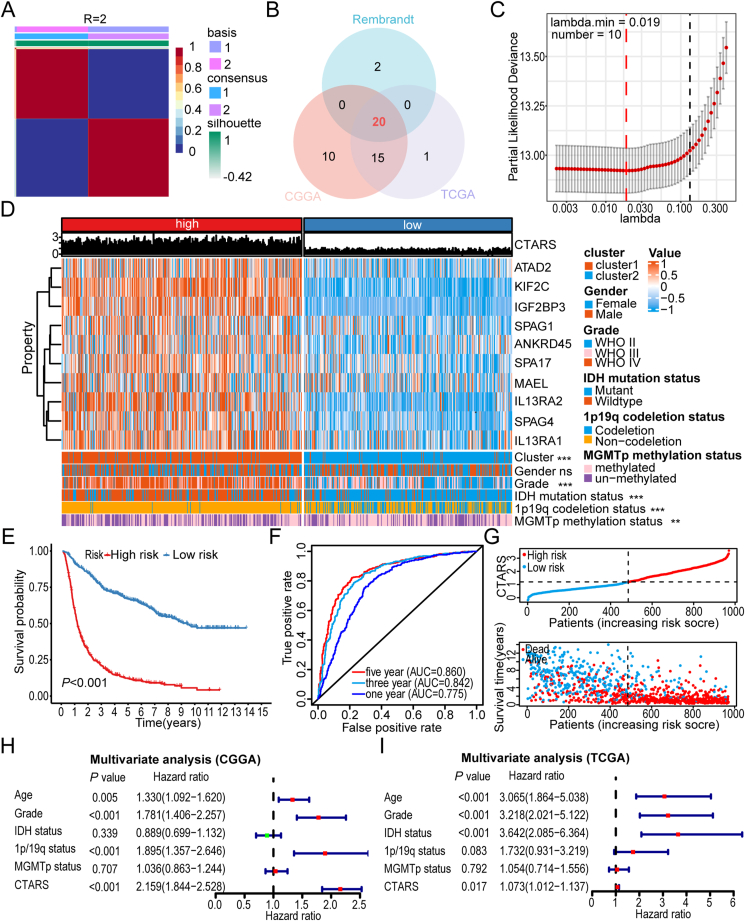
Glioma classification and risk score construction based on CTA-related gene expression. **(A)** Non-negative matrix factorization clustering matrix for rank 2 in the CGGA cohort. **(B)** The Venn diagram illustrates the overlap of the CTA genes associated with a poor prognosis across the three cohorts. **(C)** The regularization path of the least absolute shrinkage and selection operator (LASSO) demonstrates the use of cross-validation to select optimal tuning parameters. **(D)** The heatmap illustrates the relationship between CTARS and the expression levels of 10 selected genes, as well as clinicopathological characteristics. It also highlights the differences in CTARS under different clinicopathological characteristics. The Wilcoxon rank-sum test was employed for statistical analysis, with significance levels indicated as follows: ∗∗, *P* < 0.01; ∗∗∗, *P* < 0.001. **(E)** Kaplan–Meier survival curves based on the median CTARS (Log-rank test). **(F)** Receiver operating characteristic curve to predict the sensitivity and specificity for 1-, 3- and 5-year survival. **(G)** Ranked dot and scatter plots display the distribution of CTARS and patient survival status. **(H)** and **(I)** Forest plots depict the results of multivariate Cox regression analyses, evaluating the association of clinicopathological characteristics, CTARS, and overall survival in the CGGA and TCGA cohorts.

A comprehensive flowchart of the studies in this article is illustrated in Figure 1. In the CGGA cohort, gene expression profiles from 1018 samples were subjected to univariate Cox regression analysis of 93 CTA-related genes to identify 69 genes associated with OS for sample NMF clustering. For k = 2, both the cophenetic correlation coefficient and silhouette coefficient approached 1, indicating strong consistency, compactness, and separation in the clustering results (Fig. S1). The heatmap matrix showed distinct and sharp boundaries between the two clusters, demonstrating the stability and robustness of the sample clustering (Fig. 2A; Fig. S2A–S2D). t-SNE analysis further confirmed the distribution of clusters, with a close alignment to the two-dimensional t-SNE visualization (Fig. S2E). Survival analysis indicated significantly shorter OS for patients in cluster 1 than for those in cluster 2 (Fig. S2F). A chi-square test analysis revealed significant differences in clinical and molecular characteristics between the two glioma clusters, including age, grade, IDH status, 1p/19q status, and MGMTp status (Table S2). These findings suggest that gliomas can be classified into two distinct subtypes based on the clustering of CTA-related genes.

### Development of CTARS and its clinical prognostic significance in gliomas

Univariate Cox regression analyses across the three cohorts identified genes with elevated expression associated with poor outcomes, leading to the selection of 20 genes for further examination (Fig. 2B). The LASSO-Cox regression algorithm was applied to refine this list to 10 genes, which minimized the risk of overfitting (Fig. 2C). Heatmap analysis revealed significantly higher expression of these genes in the high CTARS group (Fig. 2D). Comparative assessments indicated that elevated CTARS values in groups were associated with unfavorable prognoses, such as Cluster 1, WHO high-grade, IDH wild-type, 1p/19q non-codeletion, and MGMTp unmethylation (Fig. 2D).

Kaplan–Meier curve analysis demonstrated that the OS of the high CTARS group was significantly lower than that of the low CTARS group (Fig. 2E; Fig. S3A and S3B). Time-dependent ROC curve analysis confirmed the high accuracy of CTARS in predicting OS (Fig. 2F; Fig. S3C and S3D). The risk curve and survival distribution map illustrated a direct correlation between increased CTARS, decreased survival times, and higher mortality rates among patients with glioma (Fig. 2G; Fig. S3E and S3F). Additionally, CTARS emerged as an independent risk factor for OS prediction in the CGGA and TCGA cohorts (Fig. 2H, I), with consistent results in the Rembrandt cohorts, reinforcing its role as an independent prognostic indicator (Fig. S3G).

Combining the independent prognostic indicators from the three cohorts, a personalized nomogram was developed using the Cox regression model to predict the 1-, 3-, and 5-year OS for patients with glioma. The concordance index (C index) for the CGGA cohort was 0.77. The nomogram shows the individual scoring of four survival risk factors, with cumulative scores used for OS prediction (Fig. S4A). The calibration curves demonstrated strong agreement between the predicted and actual OS (Fig. S4B). For the TCGA and Rembrandt validation cohorts, the C indexes were 0.84 and 0.69, respectively, with calibration curves confirming the robustness of the predicted and observed OS rates (Fig. S4C and S4D). These results suggest that the personalized nomogram effectively forecasts the clinical outcomes of patients with glioma. In conclusion, the newly developed CTARS, based on the expression levels of 10 CTA genes, shows significant potential for clinical application.

### Expression of the druggable target ATAD2 in glioma and its correlation with clinicopathological characteristics

**Figure 3:**
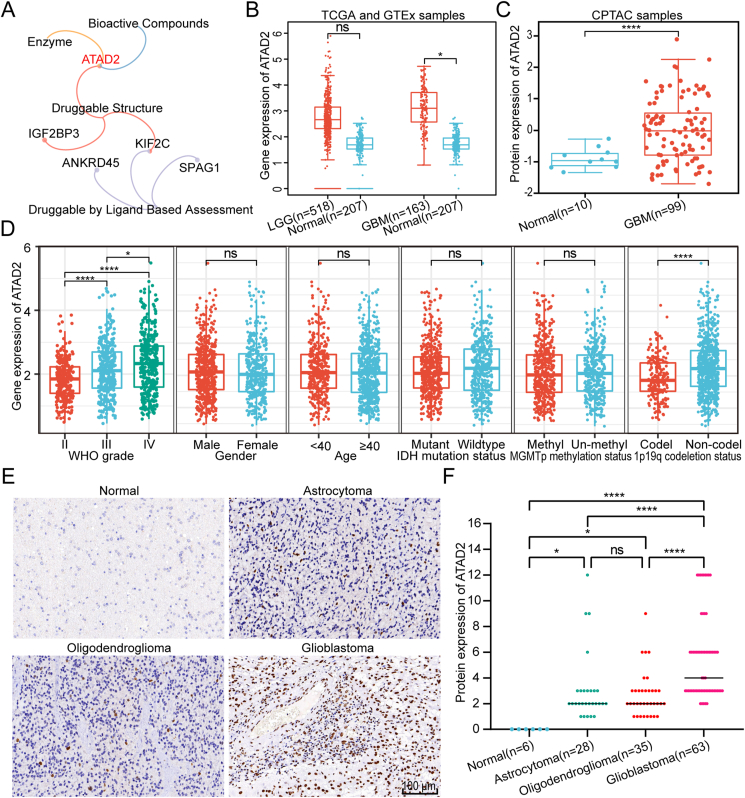
The expression of the druggable target ATAD2 in glioma. **(A)** Visualization of DepMap database-based druggability analysis of CTARS-related proteins using a network Venn diagram. **(B)** Analysis using the GEPIA database analysis revealed significant upregulation of ATAD2 mRNA expression in glioblastoma (GBM). **(C)** The UALCAN database analysis showed significant up-regulation of ATAD2 protein in GBM. **(D)** The expression of *ATAD2* mRNA across different clinicopathological characteristics was analyzed in the CGGA cohort. Statistical analyses utilized the Kruskal–Wallis *H* test followed by post hoc Dunn's test for WHO grade comparisons and the Wilcoxon rank-sum test for other parameters. **(E)** Representative cases showing immunohistochemical staining across various clinicopathological tissue types of gliomas. Scale bar: 100 μm. **(F)** Statistical analysis of the IHC scores based on the staining intensity and the percentage of positive cells. Statistical analysis was performed with the Kruskal–Wallis *H* test and by post hoc Dunn's test. Significance levels are indicated as follows: ns, not significant; ∗, *P* < 0.05; ∗∗∗∗, *P* < 0.0001.

To identify reliable therapeutic targets for glioma, a druggability assessment was performed on ten CTAs within the CTARS using the DepMap database. This assessment revealed that the ATAD2 enzyme has bioactive compounds and a druggable structure (Fig. 3A). Given the considerable druggability potential of *ATAD2* and the availability of several selective inhibitors, it was chosen for further investigation. Analysis of *ATAD2* mRNA and protein expression was carried out to compare glioma tissues with normal brain tissues. Data from the GEPIA database revealed showed mRNA levels in low-grade glioma (*n* = 518) and GBM (*n* = 163) compared to normal brain tissue (*n* = 207), with particularly marked increases in GBM samples (Fig. 3B). Analysis of the UALCAN database confirmed significantly higher protein expression of ATAD2 in GBM (*n* = 99) than in normal tissue (*n* = 10) (Fig. 3C). The CGGA cohort analysis demonstrated that *ATAD2* mRNA expression increased with higher WHO grades, with a notable rise in Grade IV. Additionally, *ATAD2* mRNA levels were significantly higher in the 1p/19q non-codeletion group than in the 1p1/9q codeletion group (Fig. 3D).

To evaluate the expression levels of the ATAD2 protein in clinical pathological specimens, IHC experiments were conducted on normal brain and glioma tissues. Figure 3E shows ATAD2 staining in representative cases from various groups. Statistical analysis was conducted based on the cumulative scores of the staining intensities and the proportions of positive cells. The results clearly showed that ATAD2 expression was significantly higher in the astrocytoma group (*n* = 28) and the oligodendroglioma group (*n* = 35) than in the normal tissue group (*n* = 6). Furthermore, the GBM group (*n* = 63) exhibited markedly higher expression levels than both the astrocytoma and oligodendroglioma groups (Fig. 3F).

Patients were classified into low and high expression groups based on their IHC scores. This classification revealed that the high expression group comprised a larger proportion of older patients, as well as those with WHO IV status, IDH wild type, and 1p/19q non-codeletion (Table 1). These findings suggest that the druggable target ATAD2 is highly expressed in GBM and is closely associated with clinical and pathological characteristics.

**Table 1:** Distribution of clinicopathological characteristics among patients with glioma in the low and high ATAD2 expression groups.

Characteristics	Low ATAD2expression	High ATAD2expression	*P*-value
n	87	39	
Gender, n (%)			0.734
Male	44 (50.6%)	21 (53.8%)	
Female	43 (49.4%)	18 (46.2%)	
Age, n (%)			0.031
< 40	24 (27.6%)	4 (10.3%)	
≥ 40	63 (72.4%)	35 (89.7%)	
WHO grade, n (%)			< 0.001
II	35 (40.2%)	1 (2.6%)	
III	19 (21.8%)	5 (12.8%)	
IV	33 (37.9%)	33 (84.6%)	
IDH status, n (%)			< 0.001
Mutant	55 (63.2%)	8 (20.5%)	
Wildtype	32 (36.8%)	31 (79.5%)	
1p/19q status, n (%)			0.003
Codeletion	31 (35.6%)	4 (10.3%)	
Non-codeletion	56 (64.4%)	35 (89.7%)	

### ATAD2 promotes the malignant progression of glioma

**Figure 4:**
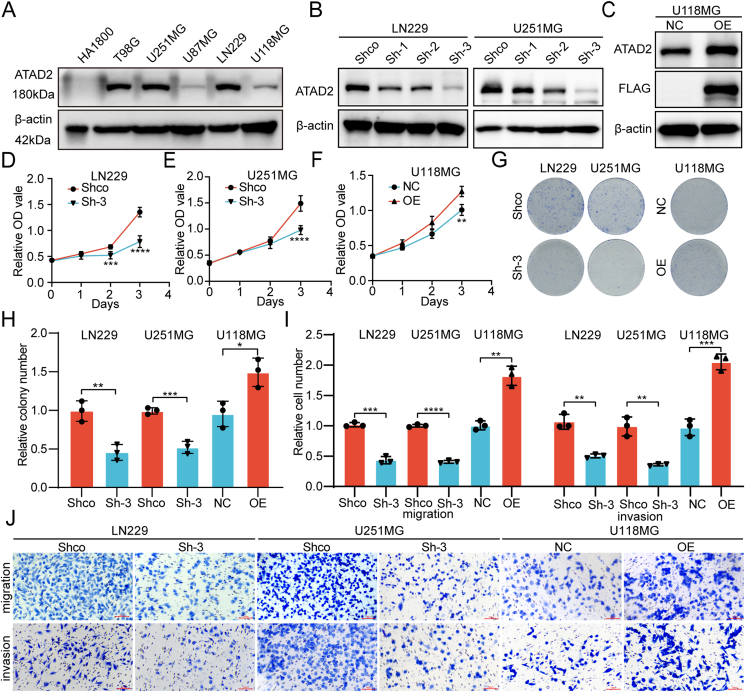
The impact of ATAD2 on the malignant phenotype of glioma cells. **(A)** The Western blot analysis shows the differential expression of ATAD2 in a normal astrocyte cell line (HA1800) and various glioma cell lines. **(B)** The Western blot analysis demonstrates the efficacy of three *ATAD2* shRNAs in reducing ATAD2 expression in the LN229 and U251MG cell lines. **(C)** The Western blot analysis revealed the expression levels of ATAD2 and the FLAG-tagged protein following ATAD2 overexpression in U118MG cells. **(D**–**F)** The CCK-8 assay shows changes in cell proliferation after knockdown of ATAD2 in LN229 and U251MG cells, and its overexpression in U118MG cells. **(G, H)** Colony formation assay and statistical analysis. **(I, J)** Migration and invasion assays and their statistical analysis. The data are presented as means ± SD. For panels (D**–**F), two-way ANOVA was performed, while unpaired two-tailed Student's *t*-tests were used for panels (H, I). Statistical significance is denoted as follows: ∗, *P* < 0.05; ∗∗, *P* < 0.01; ∗∗∗, *P* < 0.001; ∗∗∗∗, *P* < 0.0001.

To investigate ATAD2 expression in glioma cells, initial assessments were conducted using Western blotting on the normal astrocyte cell line HA1800 and various GBM cell lines. The results showed that HA1800 had negligible expression, while there was notable variability across glioma cell lines, particularly elevated levels in LN229, U251MG, and T98G (Fig. 4A). Immunofluorescence staining revealed that ATAD2 is predominantly localized in the nucleus and is more highly expressed in the LN229 and U251MG cell lines than in the T98G and U118MG cell lines (Fig. S5).

To evaluate the effects of ATAD2 knockdown on the malignant behavior of glioma cells, three shRNAs were used to generate knockdown cell lines in LN229 and U251MG. Western blot analysis showed that Sh-3 achieved the most efficient knockdown (Fig. 4B). Subsequently, ATAD2 was overexpressed in U118MG cells, which naturally have low endogenous expression levels. The Western blot results confirmed the significant up-regulation of both the FLAG-tagged protein and ATAD2 in the overexpression group (Fig. 4C). Immunofluorescence analysis revealed a marked decrease in ATAD2 expression in the Sh-3 group compared to the Shco group, while ATAD2 expression in the OE group showed a notable increase relative to the NC group (Fig. S6). The Sh-3 group, with the most effective knockdown, was chosen for further phenotypic studies. CCK-8 proliferation assays revealed a significant reduction in the OD values for the Sh-3 group compared to those of the Shco group (Fig. 4D, E), while ATAD2 overexpression led to a marked increase in the OD values (Fig. 4F). Colony formation assays demonstrated a substantial decrease in the number of colonies in the ATAD2 knockdown group, in contrast to the increase in colony formation following ATAD2 overexpression (Fig. 4G, H). Transwell migration assays indicated a significant decline in the number of migrated cells after ATAD2 knockdown, with similar results observed in invasion assays. Conversely, ATAD2 overexpression resulted in a considerable increase in migrated cells (Fig. 4I, J).

**Figure 5:**
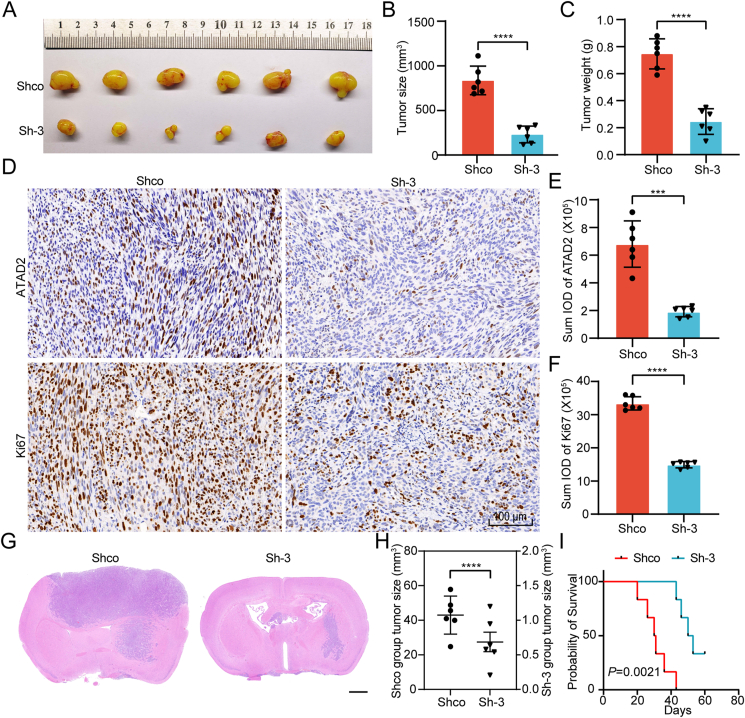
The impact of ATAD2 knockdown on Glioma tumorigenesis *in vivo*. **(A)** Gross morphological observations were conducted on subcutaneous tumors induced by LN229 cells in the Shco and Sh-3 groups of nude mice. **(B)** Statistical analysis of the subcutaneous tumor size measurements. **(C)** Statistical analysis of the subcutaneous xenograft tumor weights. **(D)** Representative images of IHC staining for ATAD2 and Ki67. Scale bar:100 μm. **(E, F)** The Image-Pro Plus software (version 6) was used to compute the sum integrated optical density of ATAD2 and Ki-67 IHC staining, followed by statistical analysis. **(G)** Representative HE staining images of intracranial orthotopic xenografts formed by LN229 cells in the Shco and Sh-3 groups. Scale bar: 1 mm. **(H)** Statistical analysis of intracranial orthotopic tumor size measurements. **(I)** Kaplan–Meier survival curve of nude mice with orthotopic intracranial tumors (Log-rank test). The data are presented as means ± SD, two-tailed paired Student's *t*-test was used to compare the results in (B), (C), (E), and (F); and two-tailed unpaired Student's *t*-test was used to compare the results in (H). Each group included *n* = 6 mice. Statistical significance is denoted as follows: ∗∗∗, *P* < 0.001; ∗∗∗∗, *P* < 0.0001.

To assess the effects of ATAD2 knockdown on tumorigenesis in mice, LN229 Sh-3 and control cell lines were used for both subcutaneous and intracranial *in situ* tumor formation experiments in nude mice. The subcutaneous tumor study demonstrated a significant decrease in tumor volume and weight in the Sh-3 group (Fig. 5A–C). IHC analyses revealed significant down-regulation of ATAD2 protein expression and the proliferation marker Ki67 in the Sh-3 group (Fig. 5D–F). Similarly, intracranial tumor formation experiments showed a notable reduction in tumor volume after ATAD2 knockdown (Fig. 5G, H). Survival analysis indicated a substantial increase in survival time for the Sh-3 group, with two nude mice surviving beyond the experimental endpoint (Fig. 5I). Collectively, these results highlight the role of ATAD2 in promoting glioma progression.

### The ATAD2-E2F1 positive feedback loop promotes PDK1 expression

**Figure 6:**
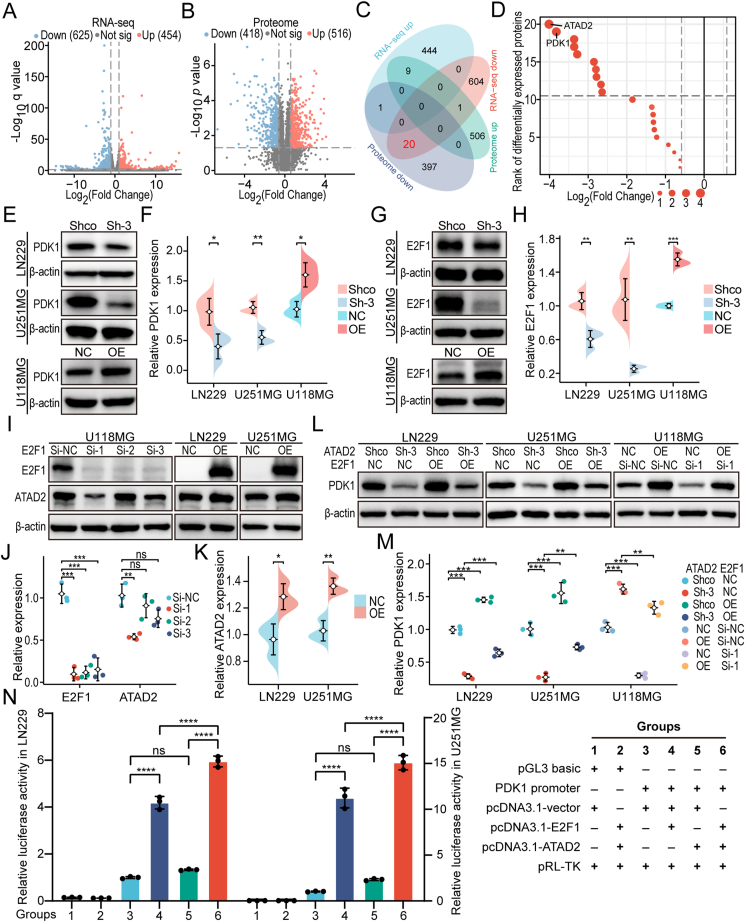
ATAD2-E2F1 positive feedback loop regulates the expression of PDK1. **(A)** Volcano plot of differentially expressed genes from RNA-seq analysis of ATAD2 knockdown and control conditions in LN229 cells. **(B)** Volcano plot of differentially expressed proteins from proteome analysis of ATAD2 knockdown and control conditions in LN229 cells. **(C)** The Venn diagram illustrates the overlap between the up-regulated and down-regulated mRNAs and proteins. **(D)** The fold change ranking plot illustrates 20 commonly down-regulated proteins. **(E, F)** Western blot analysis confirmed that ATAD2 enhances the expression of PDK1. **(G, H)** Western blot analysis confirmed that E2F1 up-regulates the expression of ATAD2. **(I–K)** Western blot analysis validated that E2F1 enhances the expression of ATAD2. **(L, M)** Western blot analysis showed that ATAD2 cooperates with E2F1 to up-regulate the expression of PDK1. **(N)** The dual luciferase reporter gene assays revealed that ATAD2 and E2F1 synergistically enhance the promoter activity of PDK1. The data are presented as means ± SD. Statistical comparisons were performed using unpaired two-tailed Student's *t*-test for figures (F), (H), and (K), and one-way ANOVA followed by Tukey's post hoc test for figures (J), (M), and (N). Statistical significance is denoted as follows: ns, not significant; ∗, *P* < 0.05; ∗∗, *P* < 0.01; ∗∗∗, *P* < 0.001; ∗∗∗∗, *P* < 0.0001.

To examine the effects of ATAD2 on the transcription and protein levels of downstream genes, RNA-seq and proteomic analysis were performed on LN229 cell lines with stable ATAD2 knockdown. Following knockdown, 454 genes were up-regulated, while 625 were down-regulated (Fig. 6A and Table S3). Proteomic analysis revealed that 516 proteins were up-regulated, and 418 were down-regulated (Fig. 6B and Table S4). A Venn diagram of the intersection of the altered genes and proteins identified 20 commonly down-regulated entities (Fig. 6C). ATAD2 and PDK1 showed the strongest reduction in expression levels based on the fold-change analysis (Fig. 6D and Table S5). To determine whether PDK1 is a downstream target of ATAD2, Western blotting was conducted, confirming a marked decrease in PDK1 expression upon ATAD2 knockdown, with a substantial increase following ATAD2 overexpression (Fig. 6E, F). Among the 20 co-downregulated proteins, several other metabolism-related proteins were identified. Three of these proteins (HK2, GLUT1, and MCT4) were selected for further validation. It was similarly observed that ATAD2 modulates the expression of these proteins (Fig. S7).

Given that previous studies have indicated ATAD2 acts as a transcriptional coactivator to enhance gene transcription,^33^ analyses utilizing the LinkedOmics database revealed substantial enrichment of the E2F transcription factor family, especially E2F1 (Fig. S8). This suggests that ATAD2 and E2F1 may collaborate in modulating the transcription of downstream genes. The literature supports ATAD2 as a downstream target of E2F1,^33^ with ATAD2 suppression impeding E2F1 expression.^34^ An investigation of the role of ATAD2 in modulating E2F1 revealed decreased E2F1 levels upon ATAD2 knockdown and a significant elevation with ATAD2 overexpression (Fig. 6G, H). To further assess the potential of E2F1 in promoting ATAD2 expression, three *E2F1* siRNAs were utilized for ATAD2 knockdown, resulting in a significant reduction, particularly in the Si-1 group, which markedly down-regulated ATAD2 expression. Conversely, E2F1 overexpression enhanced ATAD2 levels (Fig. 6I–K). These results indicate a reciprocal regulatory relationship between ATAD2 and E2F1.

Previous studies have established that E2F1 can transcriptionally regulate PDK1 expression.‍^35,36^ This finding prompted an investigation into the potential collaboration between ATAD2 and E2F1 in enhancing PDK1 expression levels. We overexpressed E2F1 in LN229 and U251MG cell lines with stable ATAD2 knockdown. Our results demonstrated that PDK1 levels in the Shco + E2F1-OE group were significantly higher than those in the Shco + E2F1-NC group. The Sh-3 + E2F1-OE group showed a notable increase in PDK1 compared to the Sh-3 + E2F1-NC group. However, the levels did not return to baseline, indicating that E2F1 overexpression can partially mitigate the reduction in PDK1 caused by ATAD2 knockdown. In U118MG cells with ATAD2 overexpression, silencing *E2F1* led to a significant decrease in PDK1 expression in the NC + E2F1-Si-1 group compared to that in the NC + E2F1-Si-NC group. Similarly, the OE + E2F1-Si-1 group exhibited significantly lower PDK1 levels than the OE + E2F1-Si-NC group, although not to baseline levels. These findings demonstrate that disrupting E2F1 expression can partially reverse the elevation of PDK1 expression induced by ATAD2 overexpression (Fig. 6L, M). Collectively, these results highlight that ATAD2 and E2F1 act synergistically to increase PDK1 expression.

To determine whether ATAD2 and E2F1 promote PDK1 transcription, dual-luciferase reporter assays were performed in LN229 and U251MG cells. The luciferase activity in the pcDNA3.1-E2F1 group was significantly greater than that in the pcDNA3.1-vector group. While the pcDNA3.1-ATAD2 group showed a slight increase, this increase was not statistically significant across multiple tests. Remarkably, the luciferase activity in the pcDNA3.1-E2F1 + pcDNA3.1-ATAD2 group surpassed that in both the pcDNA3.1-E2F1 and pcDNA3.1-ATAD2 groups (Fig. 6N). These results suggest that ATAD2 and E2F1 act synergistically to enhance PDK1 transcriptional activity in glioma cells.

### The clinical significance of the ATAD2-E2F1-PDK1 axis

**Figure 7:**
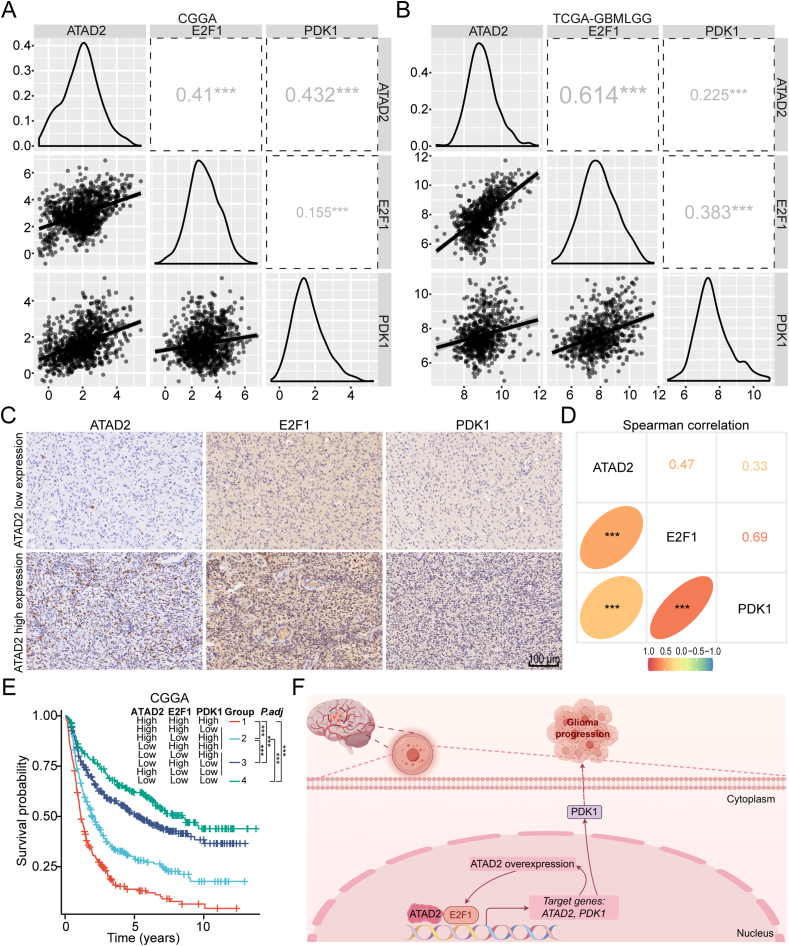
The clinical significance of the ATAD2-E2F1-PDK1 axis in glioma. **(A, B)** Analysis using the GlioVis database showed a positive correlation between the expressions of ATAD2, E2F1, and PDK1, as determined by the Spearman correlation test. **(C)** Representative cases showed the expression of E2F1 and PDK1 in glioma clinical specimens with low and high expression of ATAD2. Scale bar: 100 μm. **(D)** Spearman's correlation analysis of ATAD2, E2F1, and PDK1 expression levels in glioma clinical specimens. **(E)** Kaplan–Meier analysis and log-rank test of survival rates across distinct co-expression groups of ATAD2, E2F1, and PDK1 in the CGGA cohort. *P* values were adjusted for multiple group comparisons using the Bonferroni method. **(F)** Graphical diagram illustrating that ATAD2 promotes glioma progression and synergizes with E2F1 to increase PDK1 expression. The figure was created using Figdraw (www.figdraw.com). Statistical significance is denoted as follows: ∗∗∗, *P* < 0.001.

Given the collaborative regulatory role of the ATAD2-E2F1 positive feedback loop in enhancing PDK1 expression, we assessed the correlations among these three factors using the GlioVis database. Significant positive correlations (R > 0.15 for all pairwise comparisons) were observed among the expression levels of ATAD2, E2F1, and PDK1 in both the CGGA and TCGA-GBMLGG datasets (Fig. 7A, B). To further investigate the expression patterns of these three factors at the single-cell level in glioma, analysis of the TISCH2 database revealed that they were predominantly expressed in malignant cells (Fig. S9). We evaluated the expression levels of E2F1 and PDK1 using 126 surgical glioma specimens. Analysis of representative cases revealed a clear trend: samples with low ATAD2 expression also exhibited low levels of E2F1 and PDK1, whereas those with high ATAD2 expression showed the opposite trend (Fig. 7C). Furthermore, strong positive correlations (R > 0.3 for all pairwise comparisons) were observed among the three proteins, confirming their interrelated expression patterns (Fig. 7D).

To investigate OS variations among patients with glioma based on the co-expression of these genes, we used median expression values to categorize patients into high or low expression groups across the CGGA, TCGA, and Rembrandt cohorts. Patients were classified into four groups based on the expression levels of the three genes: Group 1 included patients with high expression of all three genes, Group 2 included those with high expression of two genes, Group 3 included individuals with low expression of two genes, and Group 4 included patients with low expression of all three genes. The analysis revealed significant differences in survival curves within the CGGA cohort, with Group 1 having the least favorable prognosis. While there was no significant difference in adjusted *P* values between Groups 3 and 4, substantial differences in OS were observed among the other groups (Fig. 7E). In TCGA, patients in Group 1 exhibited a worse prognosis compared to those in Group 4 (Fig. S10A). Similarly, in the Rembrandt cohort, patients in Group 4 had better prognoses than those in Groups 1, 2, and 3 (Fig. S10B). This pattern indicates that glioma patients with high expression of all three genes have poorer prognoses. In summary, our findings collectively demonstrate that ATAD2 promotes malignant progression in glioma and synergistically up-regulates PDK1 expression in cooperation with E2F1. These results indicate that the ATAD2-E2F1-PDK1 axis may function as a clinical prognostic biomarker (Fig. 7F).

## Discussion

Reactivated CTAs exhibit characteristics similar to those observed in gametogenesis, contributing to tumor immortality, immune evasion, invasion, and metastasis.^6^^,^^37^ A thorough investigation into the expression of CTA-related genes in glioma and their prognostic implications is warranted. This study focuses on elucidating CTA gene expression patterns and developing prognostic models for glioma. The constructed CTARS serves as an independent prognostic indicator, while a nomogram integrating age, WHO grade, 1p/19q codeletion status, and CTARS effectively predicts patient outcomes.

*ATAD2*, located on chromosome 8q24 near the oncogene *MYC*, is a notable CTA gene. Its protein structure comprises an AAA + ATPase domain and a bromodomain (BRD).^38^ ‍ATAD2 is a member of the AAA + ATPase family and is involved in diverse cellular functions. This family plays a pivotal role in ATP binding and hydrolysis, triggering conformational alterations in various substrate proteins and engaging in numerous cellular processes‍.^39^ Additionally, ATAD2 is part of the BRD family, modulating chromatin dynamics, DNA replication, and transcriptional activity by directly binding to acetylated lysine residues on histones.^38^ ATAD2 is an oncogenic protein that correlates with the prognosis of several malignancies and holds significant potential as a biomarker and therapeutic target.^12^ Current ATAD2-targeting approaches, including potential therapeutic strategies such as monoclonal antibodies, RNA-based therapies, and small-molecule inhibitors, provide new opportunities and directions for cancer therapy research.^40^ Our findings indicate that ATAD2, a druggable target, is highly expressed in GBM. This confirms its oncogenic role through both *in vivo* and *in vitro* analyses.

A comprehensive analysis utilizing transcriptomics and proteomics confirmed that ATAD2 regulates the expression of the essential metabolic enzyme PDK1. Numerous studies have highlighted the involvement of ATAD2 in various tumor metabolic processes. In melanoma, ATAD2 interacts with SOX10 and c-Myc, serving as a crucial gene.^13^ ATAD2 binds to c-Myc, enhancing the transcriptional activity of c-Myc on downstream targets and promoting aggressive cancer progression.^33^ In hepatocellular carcinoma, ATAD2 modulates the expression of kinesin family member 15, which interacts with phosphoglycerate dehydrogenase to prevent its proteasomal degradation.^41^ Numerous studies have confirmed the significant role of PDK1 in GBM. Down-regulation of PDK1 and epidermal growth factor receptor (EGFR) via RNA interference reduces PDK1-EGFR activation and HIF-1α expression, shifting the metabolic profile from the Warburg effect to oxidative phosphorylation, thereby inhibiting GBM proliferation.^42^ *In vivo* studies demonstrate that the PDK1 inhibitor dichloroacetate (DCA) suppresses PDK1 expression, EGFR phosphorylation, and tumorigenicity in GBM cells.^42^ Notably, in a clinical trial involving five patients with GBM, three showed tumor regression on MRI scans after DCA treatment, while four maintained stable disease at 15 months and remained alive at the 18-month follow-up.‍^43^ We observed that ATAD2 could also regulate the expression of metabolism-related proteins, including HK2, GLUT1, and MCT4. Collectively, these findings suggest that ATAD2 can regulate the expression of multiple metabolism-related proteins, indicating its potentially significant impact on the metabolic pathways in glioma.

As a transcription factor co-activator, ATAD2 partners with various factors, including the androgen receptor, E2Fs, and c-Myc, to facilitate tumor progression.^12^ Notably, E2F1 enhances cell proliferation by binding to promoter regions of genes essential for cell cycle regulation and DNA replication, creating an “addiction” to this multifaceted transcription factor in glioma prognosis.^44^ The degrader dBET6 targets bromodomain and extraterminal (BET) family proteins, disrupting the transcriptional program co-activated by BET proteins and E2F1. This inhibits proliferation, self-renewal, and tumorigenicity in GBM cells.^45^ Additionally, the ATPase-containing AAA + protein Pontin interacts with E2F1, significantly boosting its transcriptional activation in GBM through an ATPase domain-dependent mechanism.^46^ Previous studies have shown that E2F1 also regulates PDK1 expression. The non-SMC condensin II complex subunit D3 elevates E2F1 levels and enhances its recruitment to the promoter regions of *PDK1* and *PDK3*, inhibiting pyruvate dehydrogenase activity and the tricarboxylic acid cycle in colorectal cancer.^35^ The histone lysine demethylase KDM4A serves as a coactivator for E2F1, binding to the promoters of *PDK1* and *PDK3*, thus influencing the metabolic transition between glycolysis and mitochondrial oxidation in prostate cancer.^36^ However, the interplay between ATAD2 and E2F1 in regulating PDK1 expression in glioma is not yet fully understood. Our subsequent investigations revealed a positive feedback loop between ATAD2 and E2F1 that increases PDK1 expression and stimulates its transcriptional activity. Correlation analyses of expression and survival outcomes emphasize the critical clinical relevance of these factors. Given that ATAD2 functions as a transcriptional coactivator, it is possible that ATAD2 synergizes with other transcription factors in glioma. Future studies should investigate these potential synergistic interactions.

This study acknowledges several limitations. A larger clinical cohort is necessary to validate the accuracy and applicability of CTARS. Additionally, more *in vivo* experiments involving subcutaneous and intracranial orthotopic tumors following ATAD2 overexpression are required to strengthen the evidence for its oncogenic role. Although the regulatory mechanism of ATAD2 has been preliminarily examined, further research is needed to verify whether PDK1 mediates the oncogenic function of ATAD2 and to test the efficacy of combining ATAD2 inhibitors with DCA. Given the involvement of ATAD2 in chemotherapy resistance across various tumors,^47^^,^^48^ the observation of elevated ATAD2 levels in the temozolomide (TMZ)-resistant T98G cell line suggests a potential association with TMZ resistance. Future investigations could assess the combined efficacy of ATAD2 inhibitors and TMZ to explore their synergistic effects on chemotherapy sensitivity.

In conclusion, our study presents a comprehensive analysis of the CTA-related risk model and the druggable protein ATAD2 in gliomas. These results suggest that CTARS may serve as a valuable independent prognostic marker and that ATAD2 collaborates with E2F1 to co-activate PDK1. These findings are expected to provide a critical theoretical basis for developing more effective treatment strategies and personalized interventions, ultimately aiming to improve the survival rates and quality of life for patients.

## CRediT authorship contribution statement

**Shenghua Zhuo:** Writing – original draft, Visualization, Validation, Software, Methodology, Investigation, Funding acquisition, Formal analysis, Data curation. **Liangwang Yang:** Writing – original draft, Visualization, Validation, Software, Methodology, Investigation, Funding acquisition, Formal analysis, Data curation. **Zhimin Chen:** Writing – original draft, Visualization, Validation, Software, Methodology, Investigation, Formal analysis, Data curation. **Shenbo Chen:** Writing – original draft, Visualization, Validation, Software, Methodology, Investigation, Funding acquisition, Formal analysis, Data curation. **Shuo Yang:** Writing – review & editing, Methodology, Investigation. **Taixue Chen:** Writing – review & editing, Methodology, Investigation. **Wen-Shu Wu:** Writing – review & editing, Supervision, Project administration, Conceptualization. **Kai Wang:** Writing – review & editing, Supervision, Project administration, Conceptualization. **Kun Yang:** Writing – review & editing, Supervision, Project administration, Funding acquisition, Conceptualization.

## Ethics declaration

Ethical approval for the use of anonymous specimens was granted from the Medical Ethics Committee of the First Affiliated Hospital of Hainan Medical University (Approval number: 2023-KYL-099). Written informed consent was obtained from adult patients prior to their participation. The study was conducted in compliance with the ethical guidelines of institutional and national research committees, following the principles of the 1975 Declaration of Helsinki. Animal experiments were conducted in accordance with the National Institutes of Health Guide for the Care and Use of Laboratory Animals and were approved by the Laboratory Animal Ethics Committee of Hainan Medical University (Approval number: HYLL-2024-113).

## Data availability

The data and codes that support the findings of this study are available from the corresponding authors or Shenghua Zhuo (zhuoshenghua@muhn.edu.cn) upon reasonable request.

## Funding

This work was supported by the 10.13039/501100001809National Nature Science Foundation of China (No. 82060456), the Finance science and technology project of Hainan province (China) (No. ZDYF2022SHFZ088), the Hainan Province “South China Sea Rising Star” Science and Technology Innovation Talent Platform Project (China) (No. NHXXRCXM202351), the project of Hainan Provincial Postdoctoral Science Foundation (China) (No. 395995), the Hainan Provincial Natural Science Foundation of China (No. 821MS137), and the Key Research and Development Special Fund of the Hainan Provincial Department of Science and Technology (China) (ICAC, Grant No. SQ2025SLYZL0001).

## Conflict of interests

W.S. Wu is an editorial board member for *Genes & Diseases* and was not involved in the editorial review or the decision to publish this article. All the authors declare that there are no competing interests.
