# Supplementary material for: Druggable target ATAD2 enhances the malignant progression and cooperates with E2F1 to up-regulate PDK1 expression in glioma
Source: Genes Dis. 2025 Aug 14;13(1):101810. doi: 10.1016/j.gendis.2025.101810 (PMC12555781; doi:10.1016/j.gendis.2025.101810)
Supplement: Multimedia component 1 [file mmc1.docx]

**SUPPLEMENTARY FIGURES FOR**

**Druggable Target ATAD2 Enhances the Malignant Progression and Cooperates with E2F1 to Upregulate PDK1 Expression in Glioma**


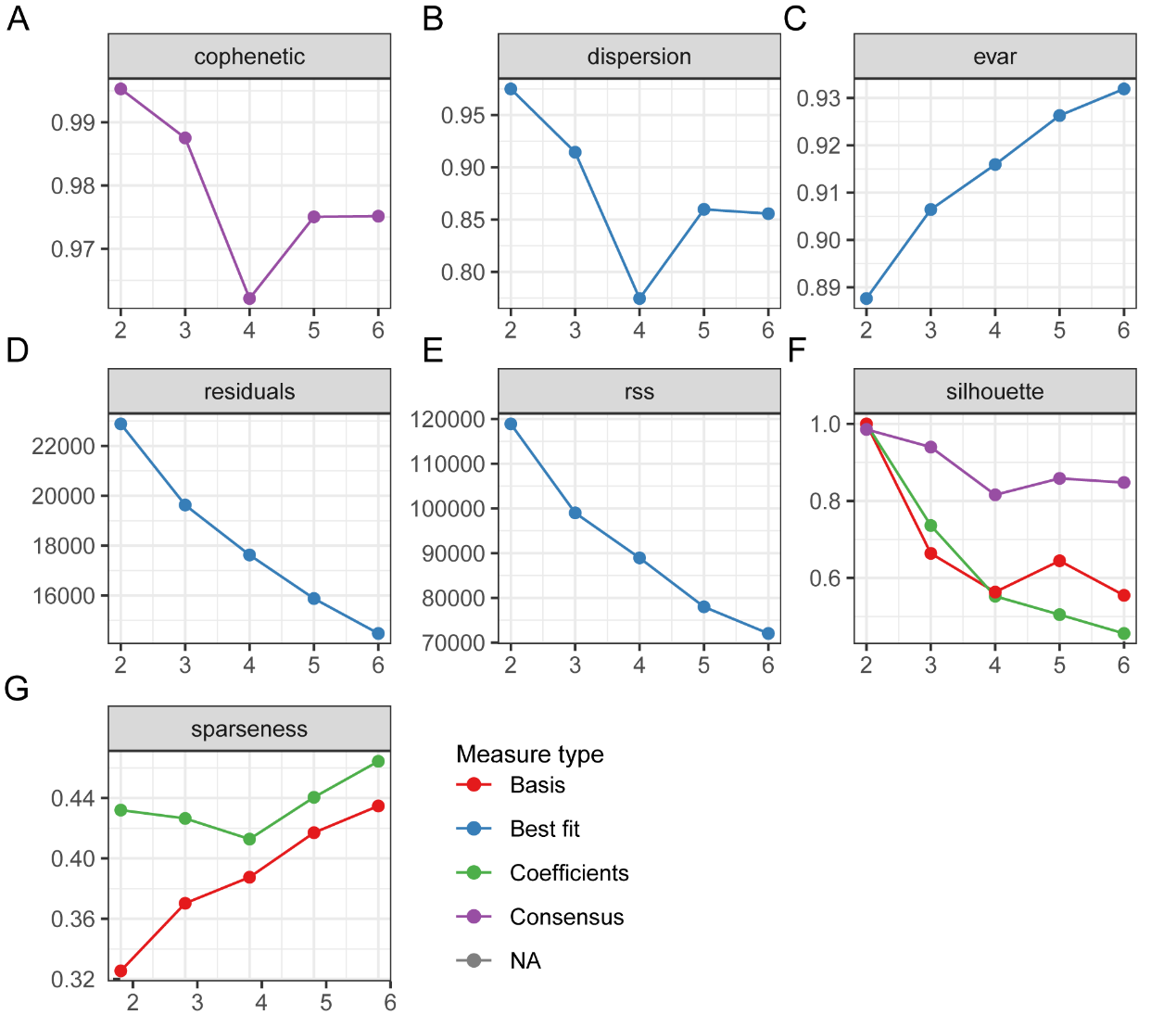


**Figure S1** The relationship between the number of clusters and the evaluation indicators. **(A)** Cophenetic correlation coefficient. **(B)** Dispersion. **(C)** Explained variance. **(D)** Residual. **(E)** Residual sum of squares. **(F)** Silhouette coefficient. **(G)** Sparseness.


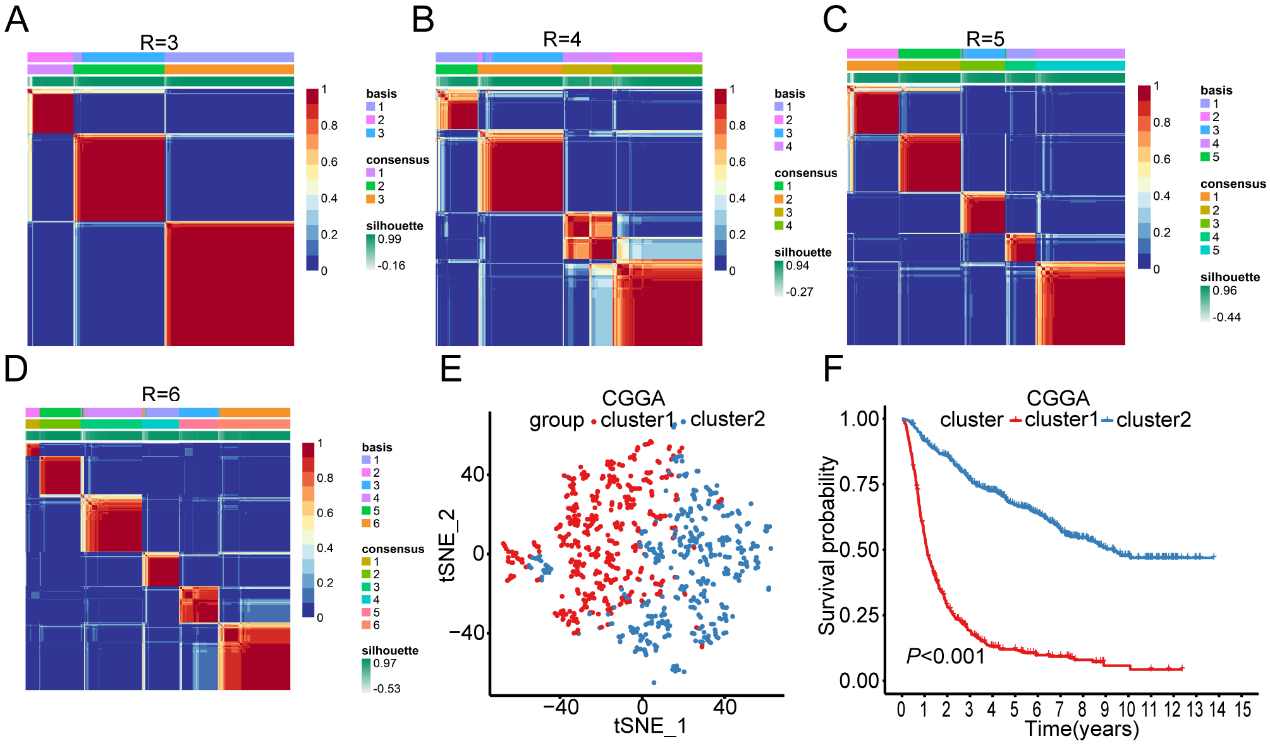


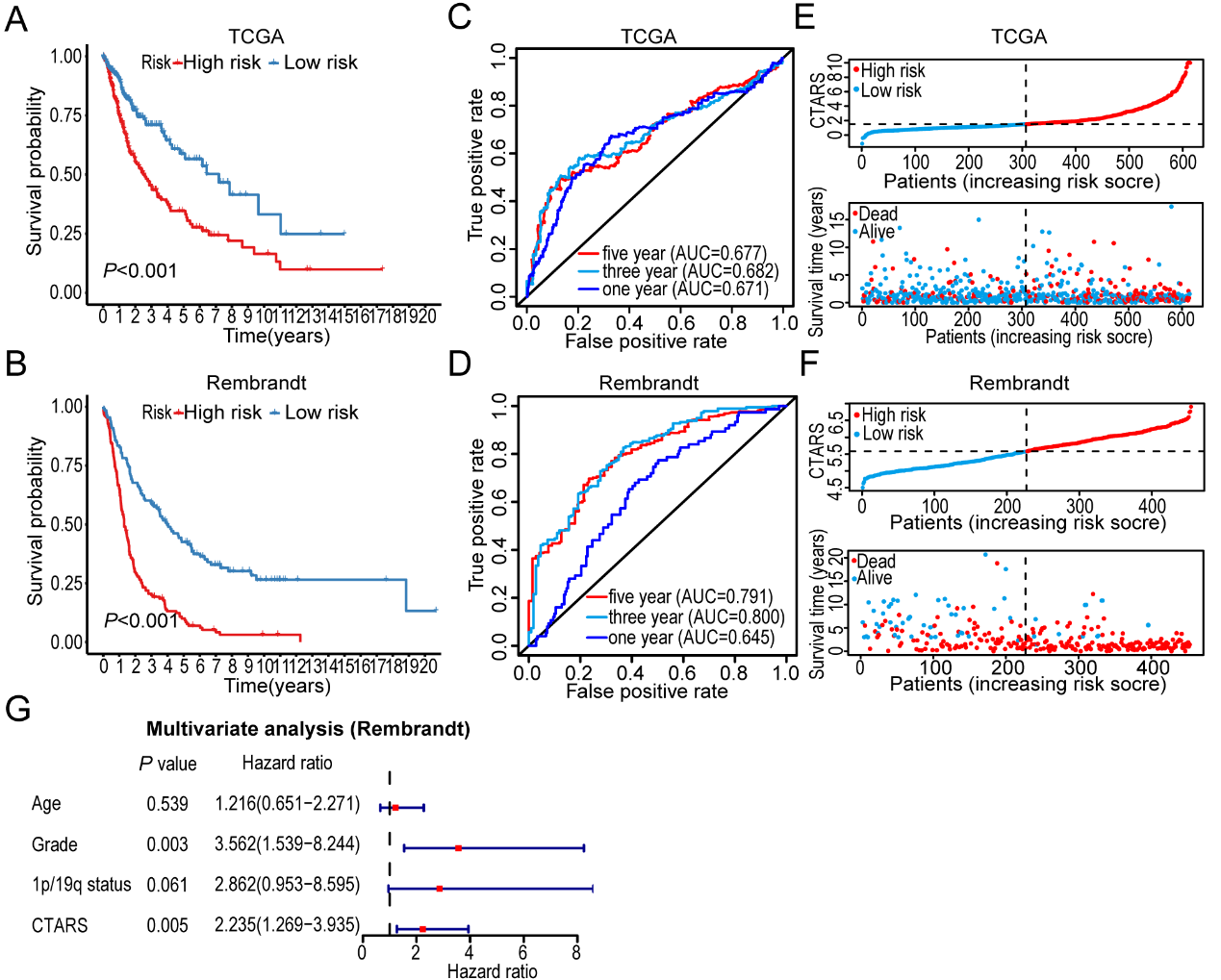
**Figure S2** Matrix of clusters in the CGGA cohort and t-SNE and survival analysis of the two clusters. **(A-D)** Cluster matrix for ranks 3 to 6. **(E)** t-SNE distribution for cluster 1 and 2. **(F)** Kaplan-Meier survival curves of two clusters (Log-rank test).

**Figure S3** Prognostic value of the CTARS in the TCGA and Rembrandt validation cohort. **(A, B)** Kaplan-Meier survival curves based on median CTARS (Log-rank test). **(C, D)** Receiver operating characteristic curve to predict the sensitivity and specificity of 1-, 3- and 5-year survival. **(E, F)** Ranked dot and scatter plots showing the distribution of CTARS and patient survival status. **(G)** The forest plots present multivariate Cox regression analyses examining the relationships of clinicopathological characteristics and CTARS with the overall survival of glioma patients in the Rembrandt cohort.


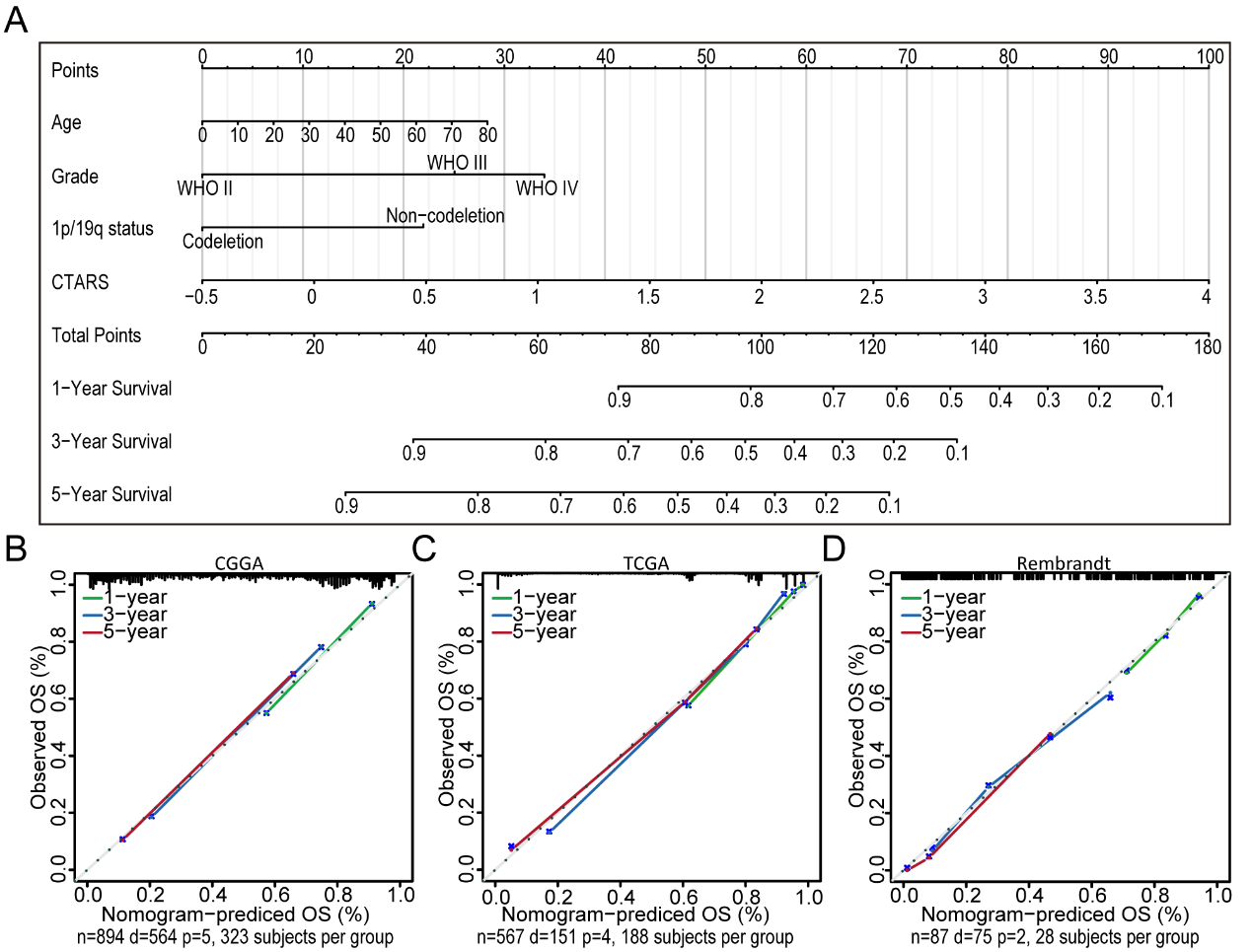


**Figure S4** Construction and validation of personalized nomogram. **(A)** The nomogram constructed based on age, WHO grade, 1p19q status and CTARS was used to predict the overall survival of patients in the CGGA cohort. **(B)** Calibration curve of the CGGA cohort. **(C)** Calibration curve of the TCGA cohort. **(D)** Calibration of the Rembrandt cohort.


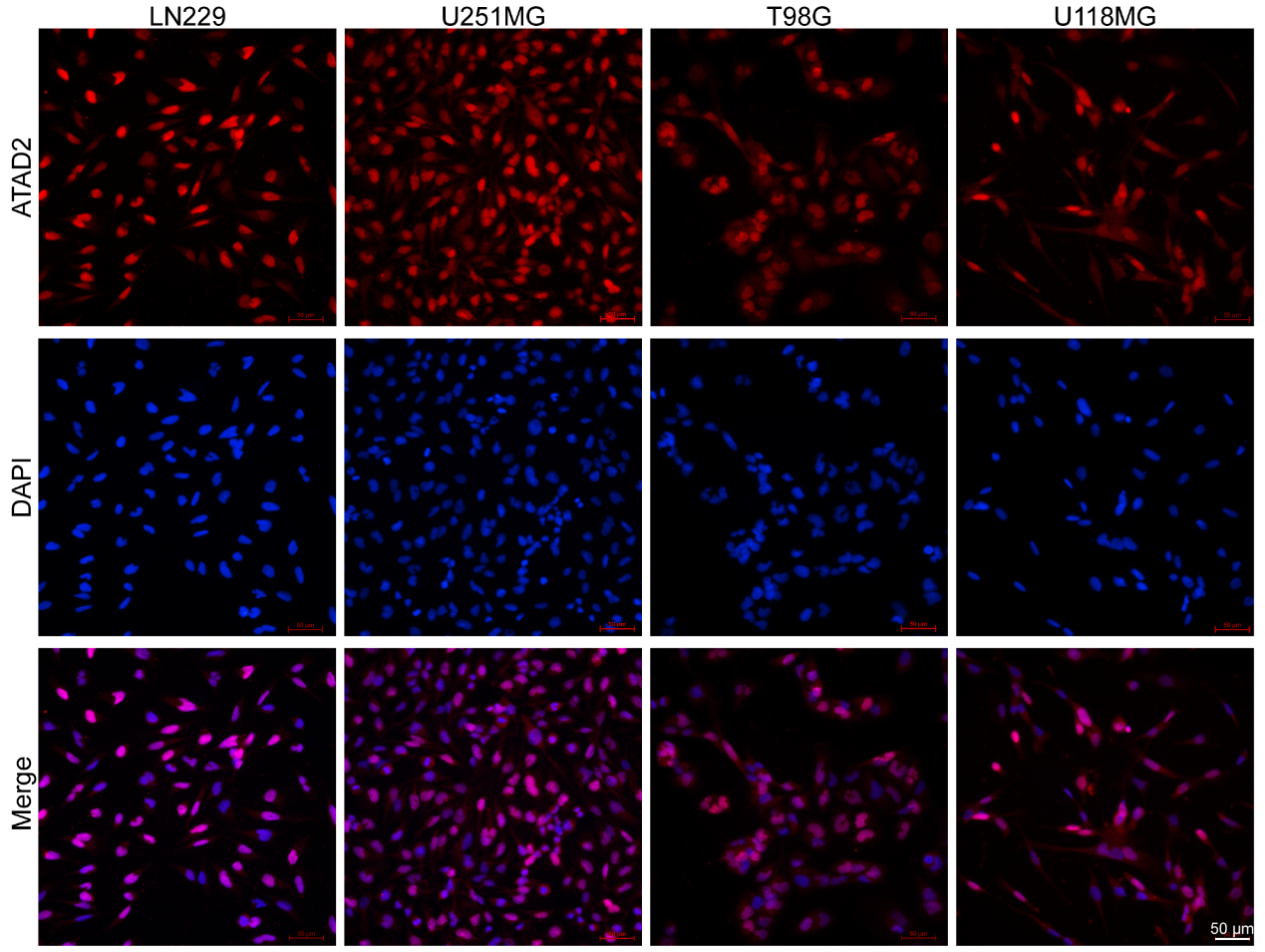


**Figure S5** Cell immunofluorescence shows that ATAD2 is expressed in the cell nucleus. Scale bars: 50µm.


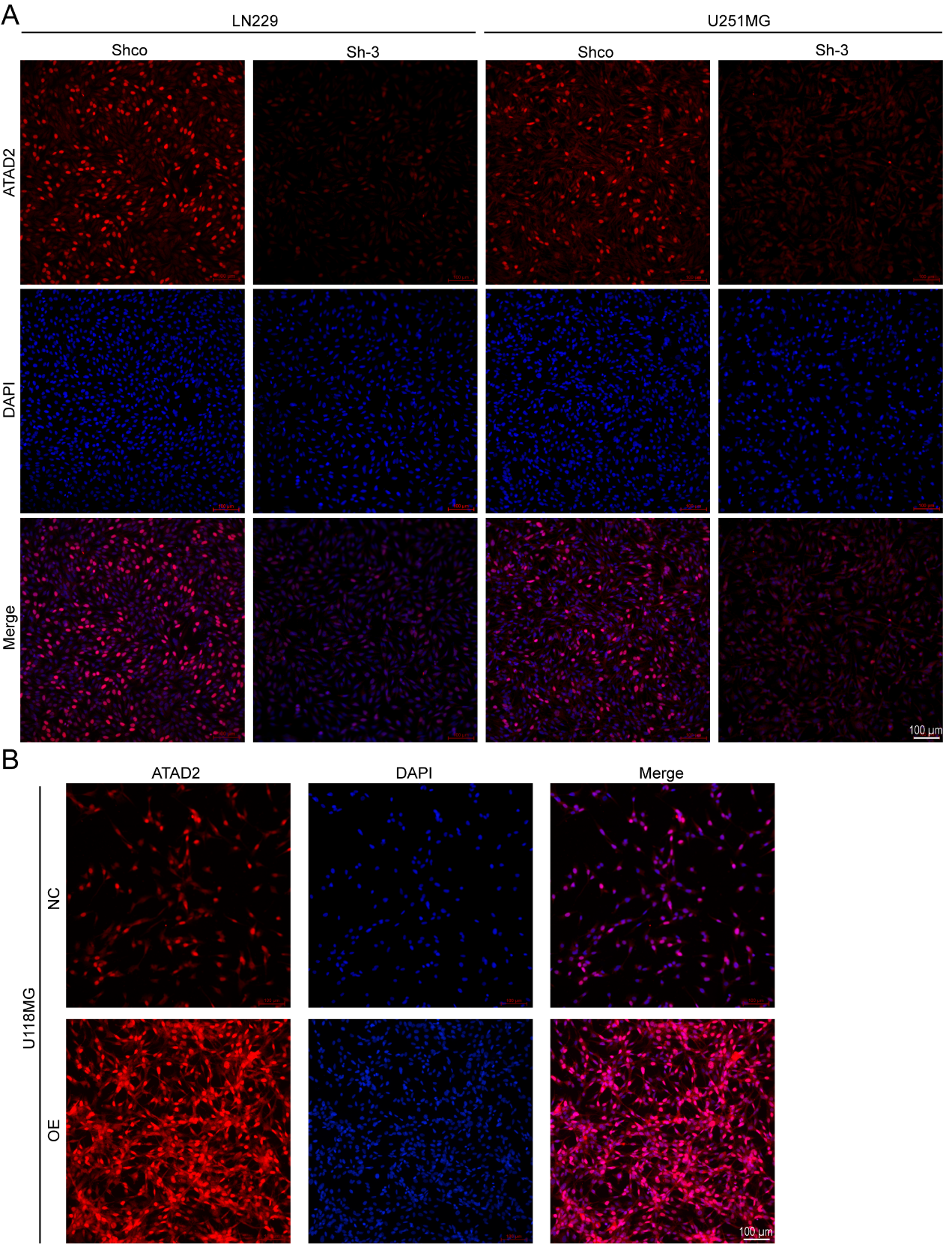
**Figure S6** Immunofluorescence assays after knockdown or overexpression of ATAD2. **(A)** Knockdown in LN229 and U251MG. **(B)** Overexpression in U118MG. Scale bars: 100µm.


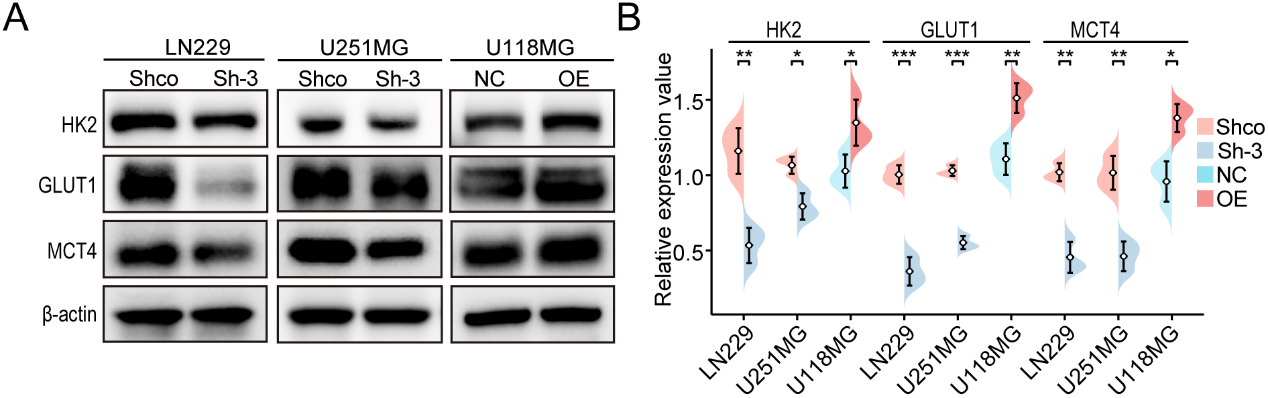
**Figure S7** Western blot analysis confirmed that ATAD2 modulates the expression of HK2, GLUT1, and SLC16A3 **(A-B)**. The data are presented as means ± SD. Statistical comparisons were conducted using unpaired two-tailed Student's t-test. Statistical significance is denoted as follows: *, *P* < 0.05; **, *P* < 0.01; ***, *P* < 0.001.


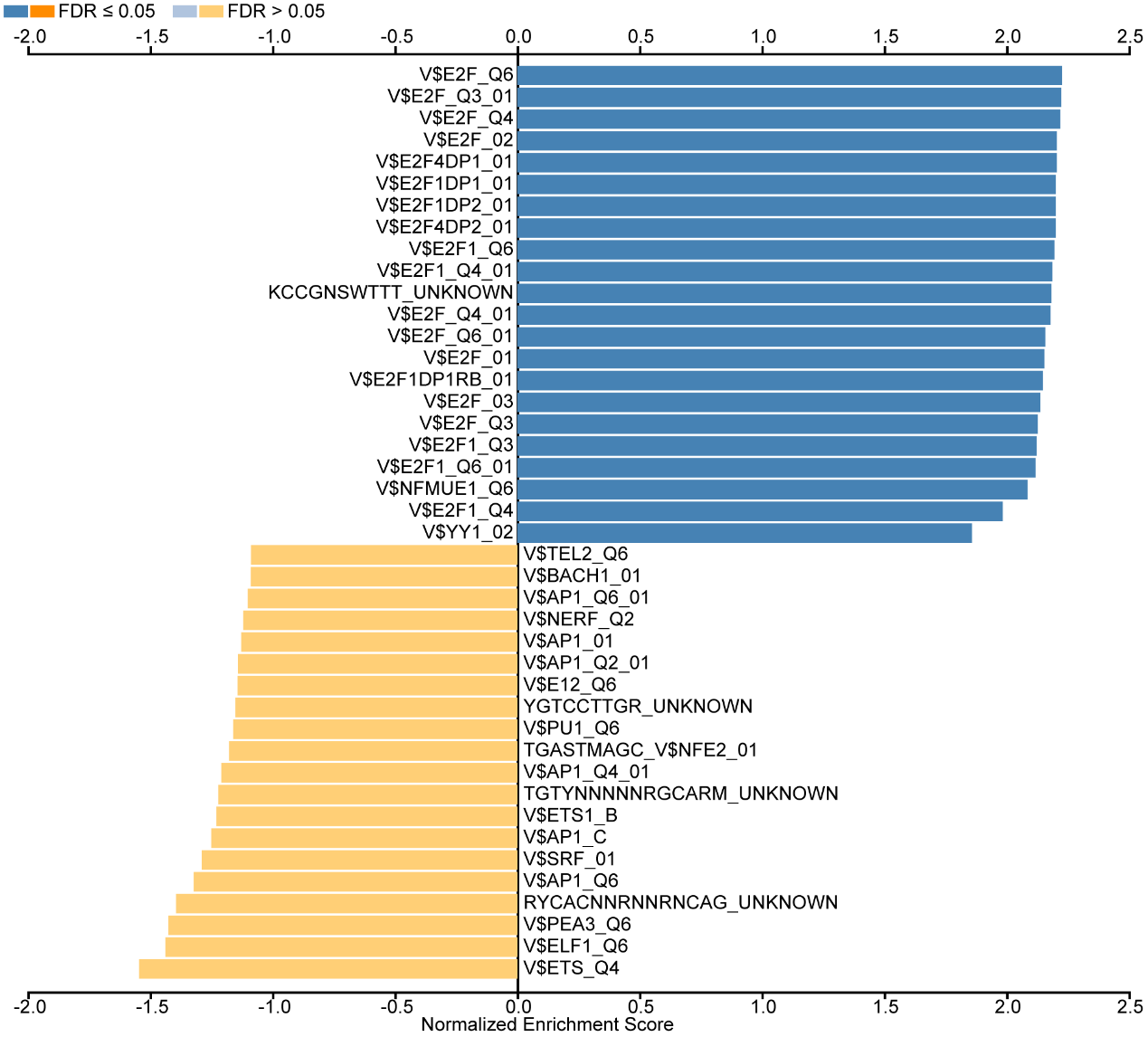


**Figure S8** Investigation of transcription factors linked to ATAD2 expression using the CPTAC_GBM dataset in the LinkedOmics database.

**Figure S9**
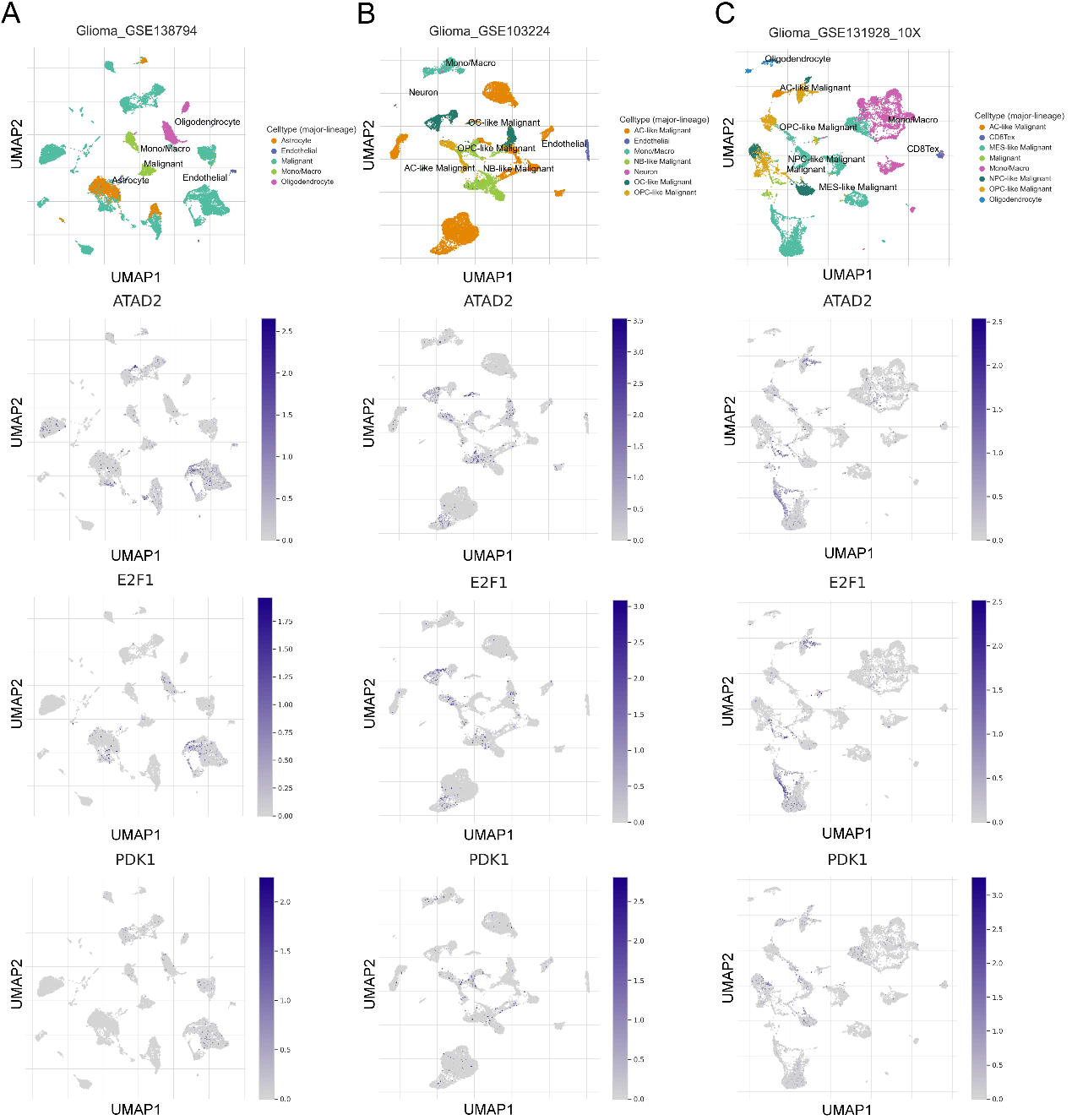
​Visualization of single-cell expression patterns for ATAD2, E2F1, and PDK1 in glioma via the TISCH2 database.​ **(A)** GSE138794. **(B)** GSE103224. **(C)** GSE131928_10X.

**Figure S**
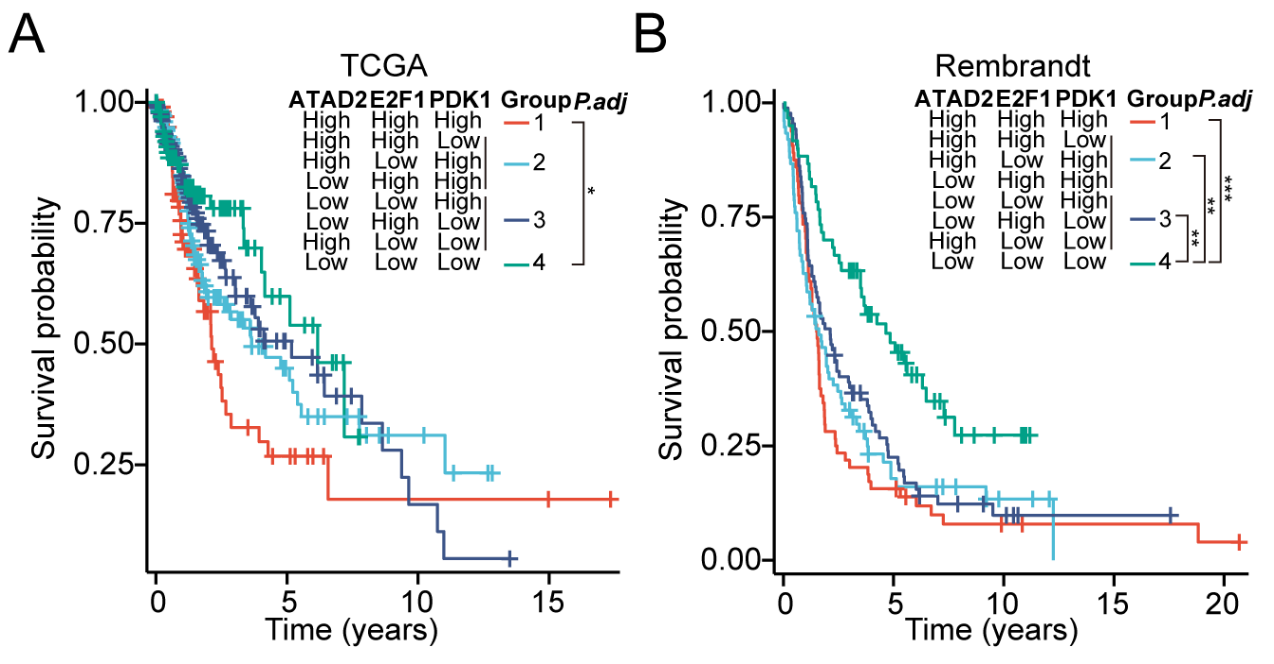
**10** Kaplan-Meier analysis and the log-rank testing of survival rates across distinct ATAD2, E2F1, and PDK1 co-expression groups in the TCGA **(A)** and Rembrandt **(B)** cohorts. *P* values were adjusted for multiple group comparisons using the Bonferroni method. Statistical significance is denoted as follows: *, *P* < 0.05; **, *P* < 0.01; ***, *P* < 0.001.
